# Digital DNA microarray generation on glass substrates

**DOI:** 10.1038/s41598-020-62404-1

**Published:** 2020-04-01

**Authors:** Johannes Wöhrle, Stefan D. Krämer, Philipp A. Meyer, Christin Rath, Matthias Hügle, Gerald A. Urban, Günter Roth

**Affiliations:** 1 University of Freiburg, Center for Biological Systems Analysis (ZBSA), Habsburgerstrasse. 49, Freiburg, 79104 Germany; 2 University of Freiburg, Department of Microsystems Engineering (IMTEK), Georges-Köhler-Allee 103, Freiburg, 79110 Germany; 3 University of Freiburg, Faculty of Biology, Biology 3, Schänzlestrasse 1, Freiburg, 79104 Germany; 4 BioCopy GmbH, Elzstrasse 27, Emmendingen, 79312 Germany; 5 University of Freiburg, Center for Biological Signalling Studies (BIOSS), Schänzlestrasse 18, Freiburg, 79104 Germany; 6 Brandenburg Medical School (MHB), Neuruppin, Germany; 7 BioCopy Holding AG, Industriestrasse 15, Aadorf, 8355 Switzerland

**Keywords:** DNA, Biochemistry, Biological techniques, DNA, Screening

## Abstract

In this work we show how DNA microarrays can be produced batch wise on standard microscope slides in a fast, easy, reliable and cost-efficient way. Contrary to classical microarray generation, the microarrays are generated via digital solid phase PCR. We have developed a cavity-chip system made of a PDMS/aluminum composite which allows such a solid phase PCR in a scalable and easy to handle manner. For the proof of concept, a DNA pool composed of two different DNA species was used to show that digital PCR is possible in our chips. In addition, we demonstrate that DNA microarray generation can be realized with different laboratory equipment (slide cycler, manually in water baths and with an automated cartridge system). We generated multiple microarrays and analyzed over 13,000 different monoclonal DNA spots to show that there is no significant difference between the used equipment. To show the scalability of our system we also varied the size and number of the cavities located in the array region up to more than 30,000 cavities with a volume of less than 60 pL per cavity. With this method, we present a revolutionary tool for novel DNA microarrays. Together with new established label-free measurement systems, our technology has the potential to give DNA microarray applications a new boost.

## Introduction

In the past, DNA microarrays have been used for a multitude of different biological applications ranging from binding experiments to gene expression analyses and genotyping^1^. Moreover, DNA arrays laid the foundation for next generation sequencing strategies^2^. Traditionally, the manufacturing of DNA microarrays is realized by one of the two different methods: microarray spotting and light-directed chemical synthesis^1^. Both methods have limitations regarding spot numbers or DNA lengths. Microarray spotting is a common method which has been in use for more than 20 years. In 1995, Schena *et al*. developed a method for printing complementary DNA to monitor the expression of multiple genes on a glass slide in parallel^3^. DeRisi *et al*. created a method to apply thousands of different DNA spots to poly-lysine coated microscope slides^4^. Good surface binding was achieved and the lengths of the different DNA samples can be chosen freely. However, a different DNA must be pre-manufactured and transferred to the slide for each spot. Consequently, washing steps need to be carried out. This is cumbersome, slows down the manufacturing process, and is cost-intensive. The distance between spots must be large in order to prevent the spots from coalescing. The initial center-to-center distance between spots was 450 μm. As a result of the development of sophisticated spotting devices, arrays with spot distances of 60–150 μm can be produced today^5,6^. The length of the DNA molecules is only restricted by PCR (up to several kb).

The second approach, light-directed chemical synthesis, was published by Fodor, the founder of Affymetrix, in 1991^7^. This approach was initially used for the generation of peptide arrays. Photolabile protecting groups which can be removed by photolithography were applied. In 1994, Pease *et al*. showed that it is also possible to generate oligonucleotide arrays via light synthesis^8^. This technology enabled the production of arrays with a very high density of different DNA spots. Chee *et al*. were able to generate DNA arrays with up to 135,000 different spots with a size of 35 × 35 μm on a single slide, using the four modified nucleotide-reagents to synthesize all different DNA sequences^9^. Today, arrays can be produced with feature sizes as small as 5 μm^10^. The disadvantage of the synthesis technology is the necessity of many photolithographic masks to synthesize the desired array. This makes the process extremely expensive and the synthesis is slow. To overcome this drawback, Nuwaysir *et al*. developed a maskless photolithography system. The light needed for the synthesis was guided to the individual spot regions via a micro-mirrors-array^11^. Even with this improvement the process is still time consuming and expensive. Moreover, the maximum DNA length is strongly restricted (typical range of 25–100 bp) because of increasing synthesis error rates. DNA microarrays can also be produced using spot synthesis. Here, the single nucleotides are individually printed onto the spots, followed by a chemical synthesis step^1,12,13^. Typically, DNA microarrays with an oligonucleotide length of 60–200 bp can be produced with this technique. Hoffmann *et al*. applied a different approach for the generation of DNA microarrays by performing a solid-phase PCR on a silicone substrate^14,15^. The main problem with this technology is that they cannot produce their arrays onto a planar glass glass surface. As they used NGS glass cavity chips as PCR reaction chambers, they needed to seal the cavities with a flexible polymer layer on the catching substrate to prevent leakage and evaporation of the reagents. Furthermore, the layer itself can be damaged or detached easily during the process. Therefore, this approach is not suitable for a standard laboratory environment. Also, with their system, they were only able to produce very few arrays at a time in a standard slide cycler. Over the last decade, microfluidics and lab-on-a-chip applications became popular for the amplification of DNA. In 2003 Liu *et al*. showed that several hundred PCR reactions can be performed simultaneously with their PDMS microfluidic matrix device, sealed with a glass chip^16^. However, the polymer based microfluidic systems is problematic in fluidic handling and the actuation of the valves in the system is challenging. With their high throughput droplet digital PCR approach Hindson *et al*. could demonstrate higher precision than with real-time PCR. They were able to quantify the nucleic acids in their sample and to accurately measure the copy number variations^17^. The problem of their system is the complex preparation to form the PCR reaction droplets and to control them in the microfluidic system. They were able to amplify and quantify the DNA, but could not generate a microarray with immobilized DNA. In our work we describe a system that can be used to produce DNA arrays with randomized spot position on standard microscope glass slides in an easy and scalable manner. We successfully generated DNA microarrays with different standard laboratory equipment and proved that our system works robustly under different conditions. We generated up to 24 arrays in a single batch with a total number of almost 100,000 PCR reaction cavities and a diameter of 150 μm per cavity. The generated microarrays can be used in conventional laboratory devices like microscopes and microarray scanners or even with new technologies like the label-free SCORE technology^18^.

## Results

**Figure 1:**
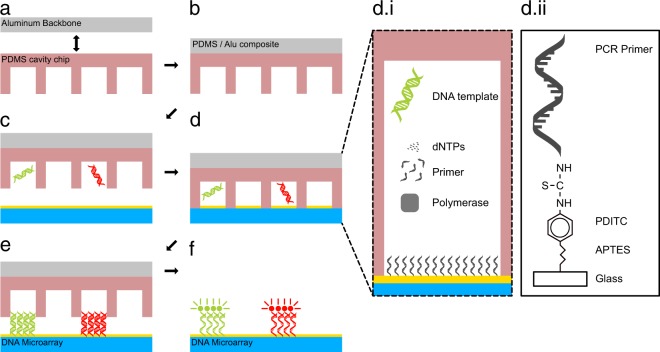
Schematic representation of the solid phase PCR process. The PDMS/aluminum composite is formed (**a**,**b**). PCR mix and DNA template are digitally filled into the cavities (**c**). The chip is sealed with the primer coupled slide (**d**). The main PCR components nNTPs, polymerase, DNA template and primer (**d.i**). In order immobilise the PCR product, the forward primer is also covalently attached on the surface (**d.ii**). After the PCR process the chip is opened and the double stranded monoclonal DNA microarray is ready for further use (**e**). For the visualization of the DNA spots, the double stranded array is dehybridized and subsequently hybridized with a fluorescently labeled probe (**f**).

We developed a system for the easy and rapid generation of DNA microarrays on standard microscope glass slides, which is based on solid phase polymerase chain reaction (SP-PCR). Therefore, we developed a PDMS/aluminum composite system to give our cavity-chips the stability to endure the harsh conditions during PCR cycling (Fig. 1a,b) (Supplementary Fig. S1). The glass slides on which the arrays are generated need to be prepared with a special surface chemistry that is able to provide binding sites of the PCR reaction products. Therefore, they are covered with PCR primers (Supplementary Fig. S2). The cavity chips are filled with PCR reaction mix in a digital dilution mode so that there is either one or no DNA template strand in each cavity (Fig. 1c). After sealing the chip and a subsequent PCR (Fig. 1d,di,dii), the final double stranded DNA array is ready (Fig. 1e). In order to visualize the DNA on the surface, fluorescently labelled DNA probes were hybridized to the DNA on the surface and scanned with a GenePix 4200A microarray scanner (Fig. 1f). We designed cavity-chips with cavity diameters ranging from 300 to 50 μm on the given area of 10 × 16 mm. The depth of each cavity is 30 μm. All cavity chips were produced with the help of a micro structured wafer, which was manufactured in a cleanroom environment and serves as a master mold. A single master mold can be used to produce several hundred cavity chips. For the proof of concept, we used a DNA-pool with two different DNA sequences with a length of 89 bp (template A) and 103 bp (template B). For temperature measurement during the PCR process, we embedded a standard PT100 sensor directly behind the array region. Delamination of the glass slide from the chips can be prevented by keeping them pressed using custom made slide holders during PCR (Supplementary Fig. S4). After the cycling process, all DNA arrays were hybridised using fluorescently labelled DNA probes, resulting in a two-colour image for each array.

**Figure 2:**
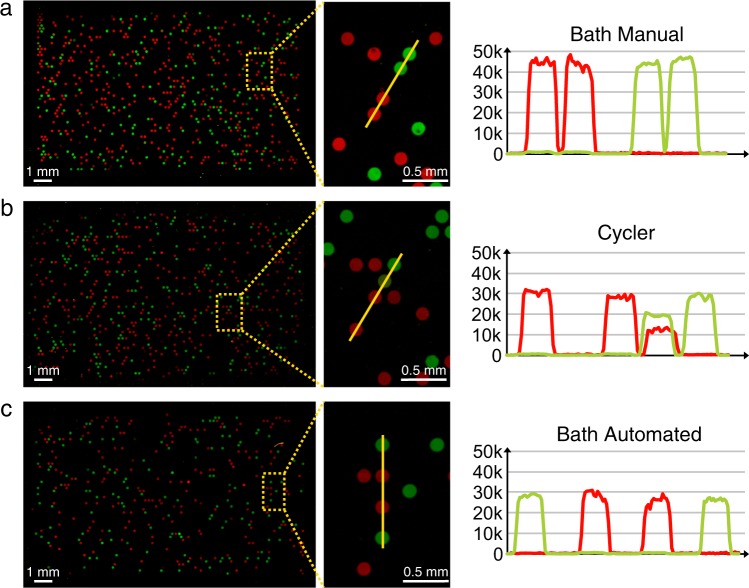
Fluorescent scans of DNA microarrays generated in three different ways. (**a**) A manually prepared array with its corresponding line scan and fluorescent signal over two red and two green spots. (**b**) An array prepared in a slide cycler can be seen. Here the corresponding line scan shows a cavity that has been filled with two DNA strands. Therefore, a red and a green signal is observed for this spot. (**c**) An array that has been prepared with an automated cartridge system. All red channels of the images were normalized to the median of fluorescent values of the green channels.

**Figure 3:**
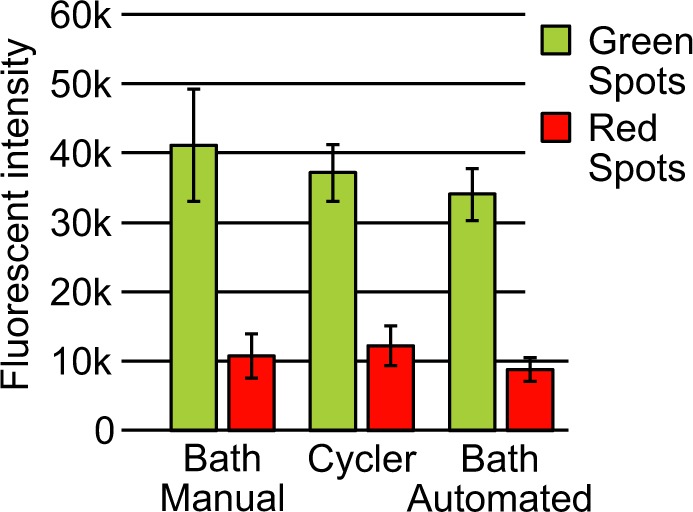
Comparison of the three different methods to generate microarrays. All green DNA spots (Template A) as well as all red DNA spots (Template B) were analyzed for all methods. The plots show the mean fluorescent values of over 13000 different spots in total (Details see Supplementary Tables S11 and S12, Supplementary Fig. S6). The results show that with all three methods DNA microarrays can be generated. Error bars represent the standard deviations.

To show the possibility that DNA microarrays can be produced via digital solid phase PCR on standard microscope slides in different ways, we established three different methods for the PCR process. First, we established a process for which the chips are manually changed from one water bath to the next to cycle them (Supplementary Fig. S8). For the second approach we used a standard slide cycler (Supplementary Fig. S9). Finally, we developed a fully automated cartridge system (Supplementary Figs. S5 and S10). For this work we prepared 31 DNA arrays in total (15 slides from automated water baths, 10 slides manually and 6 slides in the slide cycler) (Supplementary Fig. S6). The duration for a PCR differs between the methods, ranging from 90 minutes for the manual water baths method to 240 min in a standard slide cycler (Supplementary Figs. S8–10). Line scans over four representative spots were analyzed to demonstrate the homogeneity of the spots in comparison to the background signal (Fig. 2). The fact that each spot is generated in a sealed cavity leads to very sharp edges in the fluorescent values when the end of a spot is reached. Inside a single spot the fluorescent values form a plateau indicating a good homogeneity of the DNA molecules on the surface. Even double filled cavities like in Fig. 2b can be discovered. Here, the drop in the fluorescent value of the green spot in comparison to the adjacent green spot results from the co-amplification of the red DNA species in the same spot. The intensities of the different spots where analyzed with an ImageJ 1.51s macro. We analyzed over 13,000 different spots and calculated the medians for PCR cycling for each method (Fig. 3; Supplementary Tables S11 and S12). No significant differences between the different methods could be detected. This demonstrates the robustness of our cavity chip system. The fact that both, a 90-minute PCR as well as a 240 minute PCR approach yielded comparable results, shows that there is no significant decay in PCR efficiency over time. Hence, the elongation steps can be prolonged without difficulties if needed (e.g. for longer templates). The differences between the fluorescence values of green and red molecules indicate that the spPCR shows different amplification efficiencies for different DNA templates.

**Figure 4:**
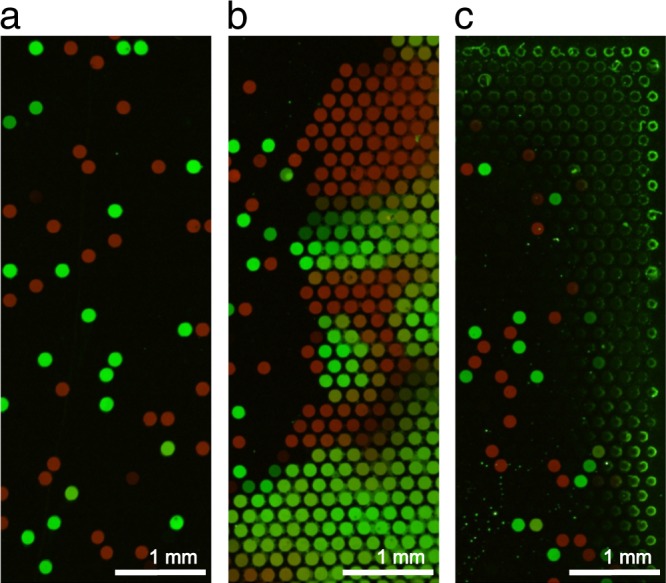
Different areas of chips after solid phase PCR. (**a**) Digital areas show where the process of the PCR worked well and no leakage or drying-ins of the cavities occurred. (**b**) Leakage can form through defects in the mold, incomplete sealing or non-uniform pressure during the PCR process. (**c**) Drying-ins can only be observed on chips which were processed in the slide cycler because of water evaporations at the chip edges.

All DNA arrays show digital areas where one full cavity is located next to an empty or different colored one (Fig. 4a). However, sometimes non-digital areas were generated (Fig. 4b). Here, many spots that are located next to each other show the same colors. This indicates a defect or leakage in that particular area. The spots located in such an area are most likely not monoclonal and therefore were not considered in the previous analysis. The main defects that can happen during the PCR process are leakages and drying-ins. Leakages are most likely to happen when the master mold is imperfect, the array is not sealed completely or not pressed during the PCR cycles. Drying-ins can be distinguished by the shape of the spots (Fig. 4c). The spots look like small donuts. The effect mainly occurs at the outer rim of some arrays that were processed in the slide cycler, because the cycler uses peltier elements to heat the slides instead of tempered water. This is why during the process the slides could face evaporation of the glycerin water solution that surrounds the array regions. If this happens, the outer rim of the arrays can run dry as well. Both effects happen accidentally and can probably be minimized by a good quality control of the chips and careful process execution. It is important to notice such defects. Affected cavities/areas should not be taken into account for the evaluation. The rest of the chip is still useable as every cavity represents a single reaction chamber and is sealed against other cavities. For this first approach we could use over 92% of the total microarray area for the evaluation (Supplementary Table S11).

**Figure 5:**
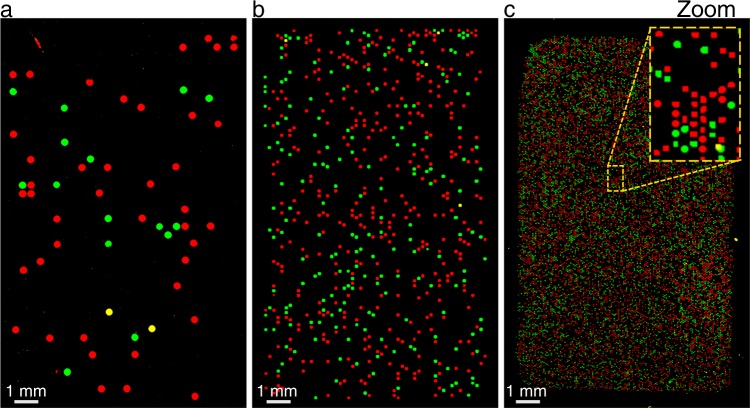
Chips with different resolutions have been developed. The array size was kept constant at 16 × 10 mm but the diameter of the cavities was varied. The chip with the lower resolution (**a**) contains 1188 cavities with a diameter of 300 μm. Our standard chip (**b**) is composed of 4104 cavities with a diameter of 150 μm each. Finally, the chip with the higher resolution (**c**) contains 31672 cavities with a diameter of 50 μm per cavity.

To demonstrate the scalability of our system we additionally developed cavity chips for the solid phase PCR process that contain lower and higher numbers of cavities (Fig. 5). The standard chips used for the experiments contain 4104 cavities within a 16 × 10 mm array region (Fig. 5b). A single cavity has a volume of about 530 pl and diameter of 150 μm. The chips with lower numbers contain 1188 cavities in the array region with a volume of 2.12 nl and a diameter of 300 μm (Fig. 5a). The chip with the highest number of cavities we used to generate DNA arrays contains 31,672 single cavities with a volume of 58.9 pL per single cavity and diameter of about 50 μm (Fig. 5c). In theory, a cavity chip could be designed with more than 350.000 spots on a single microscope slide if the whole slide area is used. The different chips were prepared in the same way as the other 31 chips within this work and the PCR was performed manually in water baths. We transformed the fluorescent scan images into binary pictures for better illustration (according to Hoffman *et al*.^14^).

**Figure 6:**
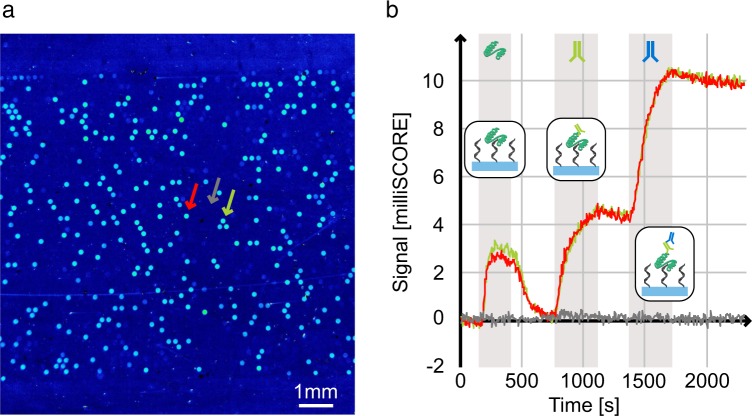
SCORE binding experiment using a generated DNA microarray. (**a**) Endpoint binding image of the DNA microarray. The brighter the signal, the more higher the binding signal is. (**b**) Detailed binding kinetic data for two DNA spots and one background spot (as indicated by arrows in (**a**)). The gray bars illustrate the time frames of analyte injections which are separated by washing steps. The analyte flushing sequence consists of a Thrombin step followed by an anti-Thrombin antibody step followed by a secondary antibody step. Furthermore, the binding steps are illustrated by small schematic pictures.

Microarrays produced by our method can also be used for high throughput, label-free binding assays using SOCRE (Single color reflectometry)^18^. A prerequisite for this is that the DNA molecules need to be immobilised directly on the glass surface, which was not the case with the previous method of Hoffman *et al*.^14^. We produced a DNA microarray using two different, well characterised Thrombin binding DNA aptamers from the literature^19^. Within our SCORE experiment, the slide was first flushed with Thrombin, followed by a primary and a secondary antibody step. Every molecular binding step was followed by a washing step. Since SCORE is an image based method, an endpoint picture illustrating the binding signals for every DNA spot was received (Fig. 6a). Moreover, we analysed and showed the detailed binding kinetic curves for two DNA spots and one background spot (Fig. 6b). Every individual binding step is visible as an increase in binding signal (milliSCORE). Furthermore, a good off-kinetic (decrease in binding signal) can be observed after the Thrombin step. In contrast to this, the off-kinetics of the antibodies are very small. The complete SCORE binding experiment together with the corresponding binding curves can also be watched and analysed as a video (Supplementary Movie S13).

## Discussion

This work describes a novel method to produce DNA arrays on standard microscope slides in an easy and scalable manner. With our approach we were able to show that the de novo generation of DNA microarrays on standard microscope glass slides with solid phase PCR is possible and can be carried out with different laboratory equipment. We show that the process of structuring dry film resists on silicon wafers via standard photolithography works well (Supplementary Fig. S7). Therefore, we are able to change the size, distance, number, location and volume of our PCR reaction chambers if needed. These wafers are used as master molds for the rapid production of the PDMS cavity chips. PDMS can provide leakage free sealing of the array region and of each single cavity. This is crucial for digital PCR as just one DNA strand can contaminate another cavity. This sealing is reversible and therefore ideal to use for our application. Our PDMS/aluminum composite is durable enough to stabilize each reaction chamber and prevent evaporation of the PCR reaction mix. Moreover, this composite system enabled us to avoid an additional PDMS layer on the glass slide^14^. With good quality management unwanted defects or artefacts (e.g. leakage, drying-ins) can even be further reduced in the future. This composite system is also suitable for integrated temperature measurements directly behind the reaction chambers. Our system withstands the harsh conditions of a solid phase PCR and still provides proper sealing after the cycling process. We were able to synthesize 15 arrays in about 120 minutes. Together with our hybridization approach, our system is simple to use within a standard laboratory environment. No high-tech equipment is needed. We believe that we could easily scale the system up to process several hundred chips at a time. Additionally, the arrays can be used as single stranded as well as double stranded arrays. As our system is able to bring microarrays to standard glass surfaces, it is also be possible to use those microarrays with new screening technologies. For instance, we were able to combine our microarray production method with the label-free SCORE technology to obtain binding kinetic information of every single spot on the array. Such a screening approach could be used for the characterisation of any DNA pool, e.g. the pool of a SELEX approach in order to find the best binding DNA aptamers. In a recent publication, we already showed that it is possible to replicate existing DNA microarrays^20^. The combination of this technology with the generation of DNA microarrays presented in this work will offer new possibilities. For instance, it would be possible to generate replica of the same DNA arrays to overcome the major challenge that every array is unique by nature. We hope that this toolbox of DNA microarray generation will make microarrays more easily accessible in standard bio labs.

## Methods

### Primer coated glass slides

Microscope glass slides were flushed with acetone, isopropanol and DI-water and were then dried in an N2 gas stream. Thereafter, slides were treated with plasma (ZEPTO, Diener electronics) at 100 W for 1 min (20 l/h gas flow). Subsequently, the slides were activated by a 30 min incubation at RT in a (3-Aminopropyl-triethoxysilane (APTES) solution (10% diH2O [v/v], 1% APTES [v/v] and 89% acetone [v/v]). Next, the slides were bathed in acetone for 5 min. This washing procedure was repeated three times. Then, the slides were dried in an N2 stream and heat treated at 110 °C for 45 min. Thereafter, the slides were cooled to RT followed by an incubation at RT for 2 h in a phenylene diisothiocyanate (PDITC) solution (10 mM PDITC in 90% DMF [v/v] and 10% pyridine [v/v]). Subsequently, the slides were rinsed with ethanol and bathed in ethanol twice for 5 min. Afterwards, slides were washed with acetone for 5 min at RT. The clean slides were dried using an N2-gas stream and vacuum treated in a desiccator for 15 min. In order to prepare the slides for SP-PCR, the surfaces were incubated in a primer solution at RT overnight (150 mM NaH2PO4, 150 mM Na2HPO4, 200 nM 5′-C6 aminated oligo [TTTTTTTTTTCCATCGCTAGACGACTCGATCAG] (Biomers, Germany)). Next, the slide surfaces were blocked (10 mg/ml BSA in Water) for 5 min. Subsequently, 5% [v/v] Ethanolamine was added to the blocking solution, followed by an incubation of another 25 min. Thereafter, the slides were flushed with DI-water and put into hot DI-water (70 °C) for 10 min. The hot water was discarded, followed by another incubation in hot DI-water (70 °C) for 5 min. Finally, the slides were flushed with DI-water at RT dried in an N2-gas stream.

### Production of cavity chip master molds

To generate the master mold for the cavity chip production, standard 4” silicon wafers were processed in a cleanroom photolithography process. For lamination of the 30 μm thick dry film resist (ORDYL SY330 Elga Europe) the laminator (Polatek, Germany) is set to the following parameters: Roll temperature 110 °C, roll distance 6.5mm, contact pressure: 3 bar, velocity 0.6 m/min, board thickness 6.6 mm. Directly before the lamination the wafer is heated on a hotplate for 60 s at 130 °C. After the lamination of the resist, the wafer needs to rest for 20 min. For exposure a mask aligner (MA6/BA6 Suss, Germany) with a foil photolithography mask is used. The masks were realized using AutoCAD (AutoCAD2014) SolidWorks (SW2013) and CorelDraw (CorelDrawX6 V16) and plotted as photo film matrix (Zitzmann GmbH, Germany). For exposure the mask aligner is set to following parameters: Illumination 12.5 s, soft contact, alignment-gap 100 μm. After lithography a post bake step at 85 °C for 60 sec is applied. To develop the resist the wafer is immersed successively in two Ordyl developer baths for 20 s and 15 s (Ordyl SY Developer, Elga Europe). Development is stopped by immersion in isopropanol and then rinsed with isopropanol followed by DI-water rinse and dried with N2 stream. The development is followed by a hardbake step at 100 °C for 60 min. To apply a non-sticky coating for later PDMS molding the wafers are placed into a desiccator and 25 μl of Trichlorosilane (Trichloro(1H,1H,2H,2H-perfluorooctyl)silane, Sigma-Aldrich) are applied onto a glass slide lying next to the wafer. After applying vacuum for 8 hours and a post bake step of 60 min at 90 °C the wafer is ready to use.

### Cavity chip production

**Figure 7:**
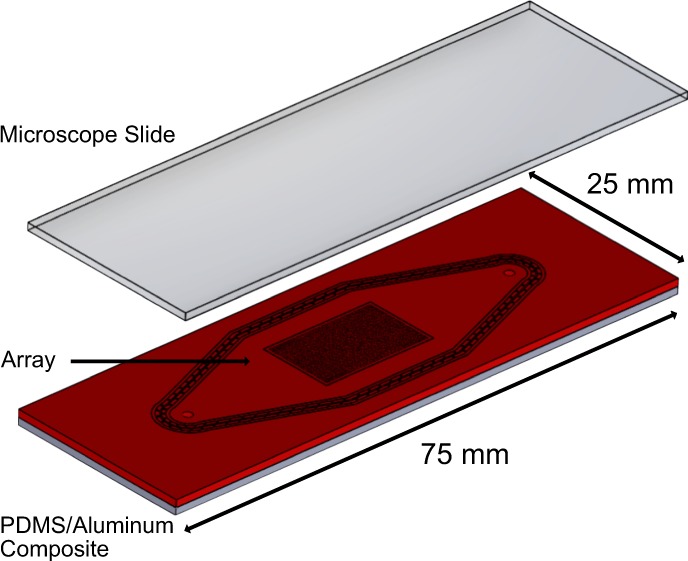
Schematic of the cavity chip system made out of a PDMS/Aluminum composite. The PDMS is bonded to the aluminum backbone and therefore has the advantage of being able to seal the array region while being stable enough for the PCR process. The array region is centralized on the chip and a standard microscope slide can be used for sealing. The chip has a thickness of 2 mm, an array size of 16 × 10 mm and yields 4104 individual cavities.

The cavity chip is a PDMS/aluminum composite. Molding tools were designed and milled in-house to keep the structured wafers and the aluminum backbones at the right location and distance from each other. The wafers are glued to the top lid and dried overnight (Soudal FIX ALL CRYSTAL). The aluminum backbones are cleaned with ethanol and activated in an O2 plasma for 2 min at 100 W and 20 l/min gas flow. After the plasma activation the backbones are placed in the bottom part of the molding tool where they are kept in position. For the activation of the PDMS (607, Wacker, Germany) the component A is mixed with the component B according to manufacturer information in a speed mixer at 1500 rpm for one minute. 9 g of the PDMS solutions is poured in every molding form directly on top of the backbones. The forms are then put in a desiccator and vacuum is applied for 6 minutes. After the vacuum step the top lid (containing the wafer with the desired cavity structures) is put on the bottom part. The assembly is heated at 60 °C for 90 min in an oven (UN 30 Plus, Memmert, Germany). After curing the PDMS/aluminum composite is disassembled from the mold and protruding PDMS is cut away with a scalpel. For fluidic connections holes are punched through the PDMS with a biopsy needle (Science Services GmbH, Germany). After that the stiff and durable composite cavity chip is ready for use (Fig. 7 and Supplementary Fig. S1).

### Solid phase PCR process

The cavity chips are filled with 5 μl of PCR reaction mix (1x Taq reaction buffer, 5 U Taq-polymerase [QIAGEN, Germany], 1.5 mM MgCl2, 0.6 mM dNTPs [QIAGEN, Germany], 0.05% Tween 80 [v/v], 3 mg/ml BSA, 0.125 μM forward DNA primer, 2 μM reverse DNA primer) and sealed with primer coupled glass slides(Supplementary Fig. S2). After the cavity region is sealed tightly the surrounding gap between the array region and the chip sealing is filled with a 50% glycerin solution via the fluidic connections to prevent expansion of enclosed air during PCR cycling and therefore delimitation of the array region (Supplementary Fig. S3). The array region does not come into contact with the glycerin mixture as this part of the chip is completely sealed before. The following DNA sequences were used: CCATCGCTAGACGACTCGATCAGTTTTTTTTTTTTTTTTTTTTGGTTGGTGTGGTTGGTCATTCGATAGTTGTTAACTTAAGCAGAGGC (template A); CCATCGCTAGACGACTCGATCAGTTTTTTTTTTTTTTTTTTTTAGTCCGTGGTAGGGCAGGTTGGGGTGACTTCATTCGATAGTTGTTAACTTAAGCAGAGGC (template B); CCATCGCTAGACGACTCGATCAG (forward primer); GCCTCTGCTTAAGTTAACAACTATCGAATG (reverse primer).

### Reaction setup SP-PCR in slide cycler

For the PCR in the slide cycler (peqSTAR *in situ* X VWR) four chips can be processed simultaneously (Supplementary Fig. S9). The lid of the cycler was customized with a spring system to keep the chips in position during PCR and to apply pressure to the array region during the cycling process. A start temperature of 23 °C was held for 60 s. For the first denaturation step 97 °C was applied for 150 s to reach over 90 °C in the chip. This step was followed by 45 3-step-cycles of PCR with a denaturation step of 97 °C for 90 s, annealing step at 58 °C for 75 s, and an elongation step at 74 °C for 75 s. The temperature profile inside the chip was checked and adjusted with an implemented PT100 (Innovative Sensor Technology IST AG) located directly under the array region and measured with a temperature logger (Testo T176).

### Reaction setup SP-PCR manually in water bath

The slide cycler is limited to process simultaneous four chips at a time. For high-throughput experiments we decided to perform the PCR quite classical in standard laboratory water baths. We used 3 water baths (CORIO CD27 Julabo, Germany) to precisely hold the three temperatures during PCR. We developed a holder system to keep 24 (or more) slides sealed and in position during water bath PCR (Supplementary Figs. S4 and S8). The PCR in the water bath must be carried out manually, so good timing of the single steps is necessary. We used an in-house programmed software to ensure an exact cycle timing. The program is open source and can be downloaded from GitHub (https://github.com/SKscience/PCR-Commander). The program parameters are as follows: 45 Cycles with denaturation for 35 s at 90 °C, primer annealing for 30 s at 59 °C and elongation at 72 °C for 50 s. The temperature profile was checked and adjusted with an implemented PT100 (Innovative Sensor Technology IST AG, Switzerland), and measured with a temperature logger (Testo T176) during the PCR process.

### Fully automated SP-PCR

For the fully automated cartridge process a Raspberry Pi 3 micro controller is used to actuate valves and a pump. For pumping a self-priming liquid ring pump CAM 80E (Linn, Germany) is used and for the electrically actuated valves (0255 series, Bürkert, Germany). For switching the 220 volts with the micro controller we use an 8 channel 5 V DC relays module (Elegoo, China). The cartridge in which the slides can be placed was designed in-house and milled out of PMMA to process 16 slides at a time (Supplementary Figs. S5 and S10). As tubing we used the Festo Pun tubing with an outer diameter of 10 mm. The protocol for the PCR was as followed: Denaturation for 45 s at 92 °C (water temperature). Primer annealing for 45 s at 58 °C (water temperature). Elongation for 65 s at 73.5 °C (water temperature). The temperature profile inside the chip was checked and adjusted with two implemented PT100 (Innovative Sensor Technology IST AG, Switzerland) at the beginning and the end of the cartridge and measured with a temperature logger Testo T176 (Testo, Germany). For PCR the temperature was additionally measured directly at the array region.

### Batchwise hybridization of microarrays

For the dehybridization of double stranded DNA after the copy process the slides are washed in 5x SSC buffer containing 0.1% SDS [v/v] for 5 min. Thereafter, the slides were incubated in 0.1xSSC buffer at RT for 5 min, followed by an incubation in dehybridization solution (50% urea [v/v], 340 mM NaCl in DI-water, 0.5% Tween 20) at 95 °C for 5 min. Next, the slides were washed in DI-water at RT for 5 min. Afterwards, the slides were put into hybridization solution (5x SSC buffer, 0.1% SDS, 10 nM Cy5 labeled DNA probe [GCCCTACCACGGACT] (Biomers, Germany), 10 nM Cy3 labeled DNA probe [CCAACCACACCAACC] (Biomers, Germany)) at 95 °C for 5 min. Afterwards, the slides within their hybridization solution were cooled down to 40 °C for 10 min. Then, the slides were incubated in 2x SSC with 0.1% SDS [v/v] at 40 °C for 3 min followed by an incubation step in 1x SSC at 40 °C for 5 min. Thereafter, slides were flushed with DI-water and dried (N2 gas stream). Finally, slides were scanned in a GenePix 4000B microarray scanner at 10 μm resolution (GenePix software version 7 Pro). The further analyses were made using ImageJ 1.51s.

### SCORE binding assay

The microfluidic SCORE experiment setup was derived from Krämer *et al*.^20^. The produced DNA microarrays were de-hybridised (50% urea [v/v], 340 mM NaCl, 0.5% Tween 20) at 95 °C for 5 min. Next, 1 ml of 10 mg/ml BSA in BBKC buffer (20 mM TRIS, 100 mM NaCl, 2 mM MgCl2, 5 mM KCl, 1 mM CaCl2, 0.02% Tween20 [v/v], pH 7.6) was directly applyed onto the array region and incubated for 10 min. Thereafter, the slides were washed with DI water to remove the blocking solution. After drying them in an N2 stream, the slides were inserted into the SCORE machine. The following microfluidic sequence was performed: [1] BBKC buffer (60 μl/min, 300 s); [2] 10 mg/ml BSA in BBKC buffer (600 μl/min, 300 s); [3] BBKC buffer (60 μl/min, 300 s); [4] 10 μg/ml Thrombin (Sigma-Aldrich, Germany) (60 μl/min, 300 s); [5] BBKC buffer (60 μl/min, 600 s); [6] 10 μg/ml anti-thrombin antibody (ab20877, Abcam, Germany) (60 μl/min, 300 s); [7] BBKC buffer (60 μl/min, 200 s); [8] 10 μg/ml secondary antibody (ab150110, Abcam, Germany) (60 μl/min, 300 s); [9] BBKC buffer (60 μl/min, 200 s).

## Supplementary information

Supplementary Information.

Supplementary Movie
