# Supplementary material for: Digital DNA microarray generation on glass substrates
Source: Sci Rep. 2020 Apr 1;10:5770. doi: 10.1038/s41598-020-62404-1 (PMC7113318; doi:10.1038/s41598-020-62404-1)
Supplement: Supplementary file 1 — Supplementary Information. [file 41598_2020_62404_MOESM1_ESM.pdf]

Supplementary File

Digital DNA microarray generation on glass substrates

Johannes Wöhrle, Stefan D. Krämer, Philipp A. Meyer, Christin Rath, Matthias Hügle, Gerald Urban, and Günter Roth

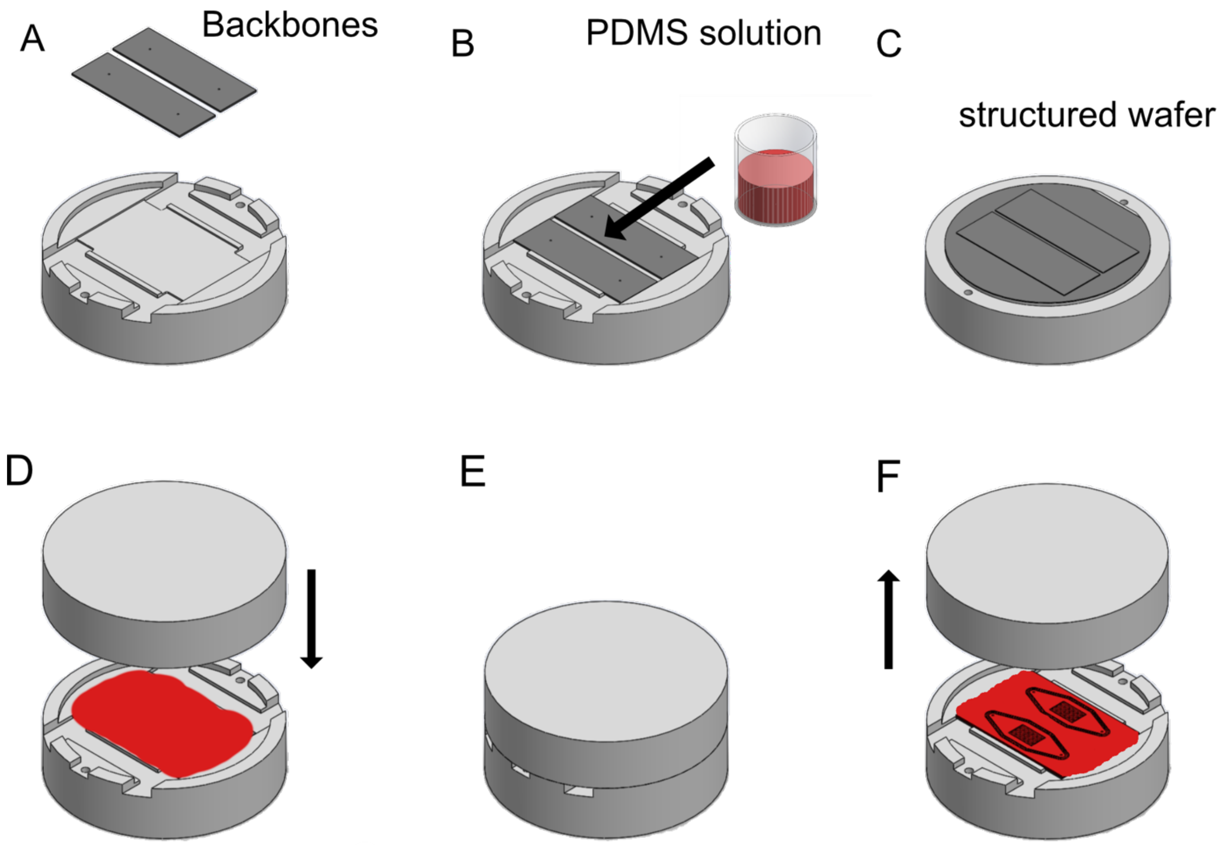

Supplementary Figure S1 Schematic of the molding process. Backbones are inserted in the bottom of the molding tool (A). Liquid PDMS solution is poured over the Backbones (B). Lid is cleaned with N_2 stream (C). Lid and bottom are brought together and closed (D/E). After the polymerization the mold is disassembled (F).

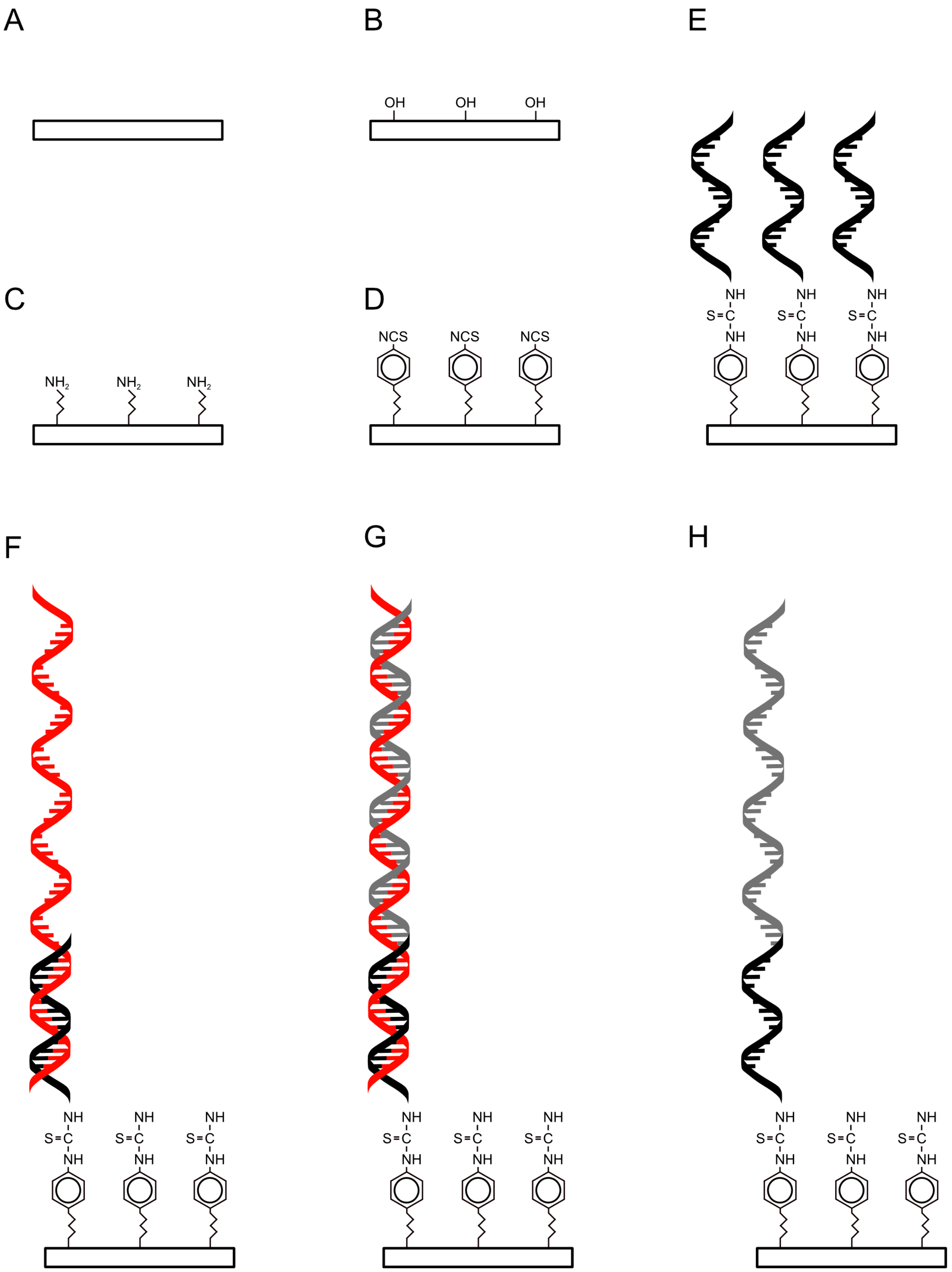

Supplementary Figure S2 Schematic of surface chemistry and solid phase PCR method. A glass substrate is cleaned and activated with oxygen plasma (A,B). The reactive OH groups at the surface are silanized with APTES (C). After the APTES activation the slide is incubated with PDITC and forms NCS groups at the surface (D). The PDITC can covalently bind aminated primer for the PCR process (E). During the PCR a single stranded DNA template binds to the primer (F) and is elongated (G). After the elongation the full length DNA strand is now covalently bound to the substrate (H).

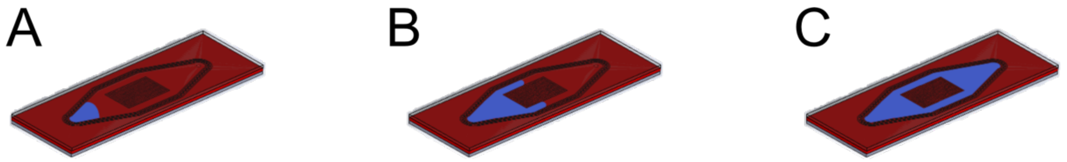

Supplementary Figure 3 Schematic of the glycerin filling process before PCR. Glycerin water mixture is flushed into the chip (A). The chip is slowly filled with the mix (B). Filling of the flow cell is stopped when the whole chip is filled (C).

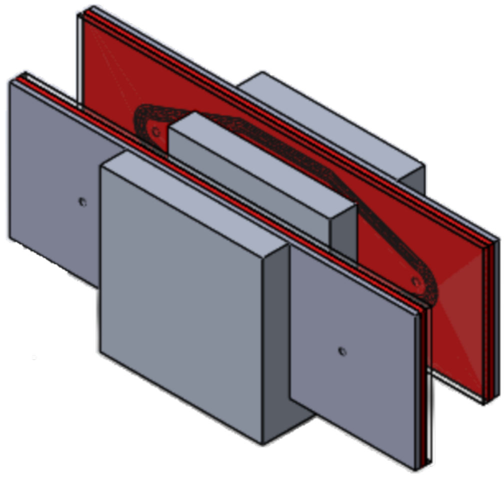

Supplementary Figure S4 Slide holder made out of aluminum for clamping of the slides during PCR process.

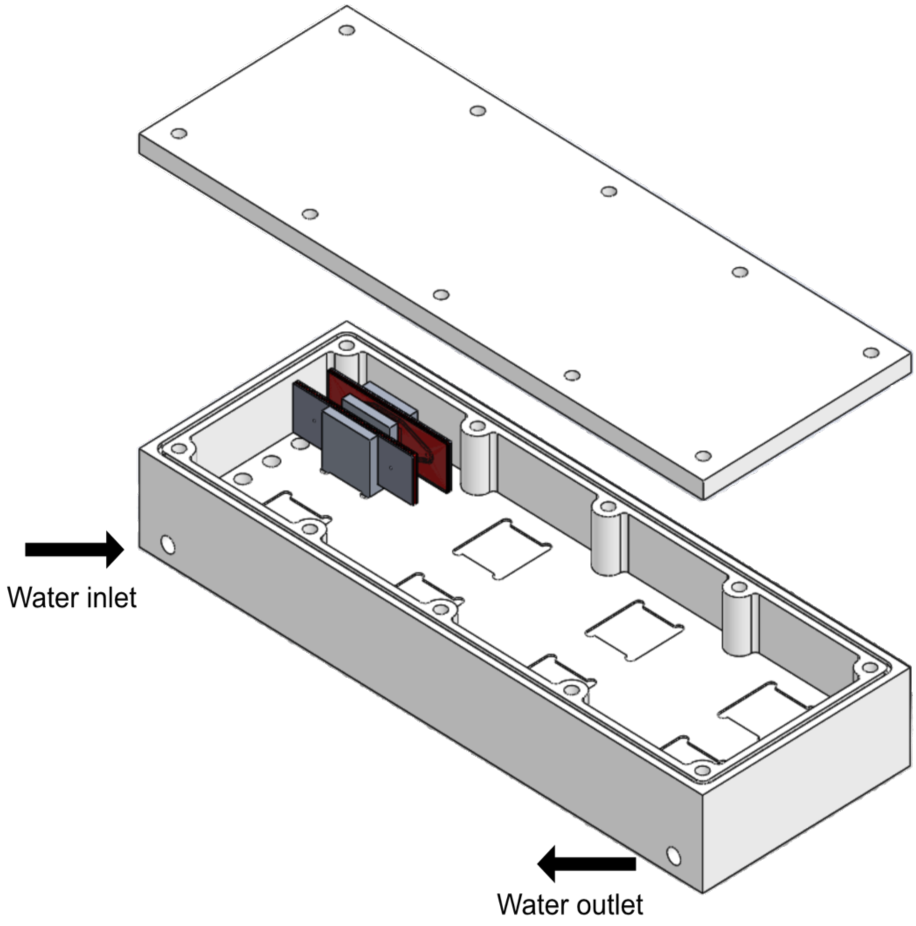

Supplementary Figure S5 Cartridge system milled from PMMA for automated cycling of the chips

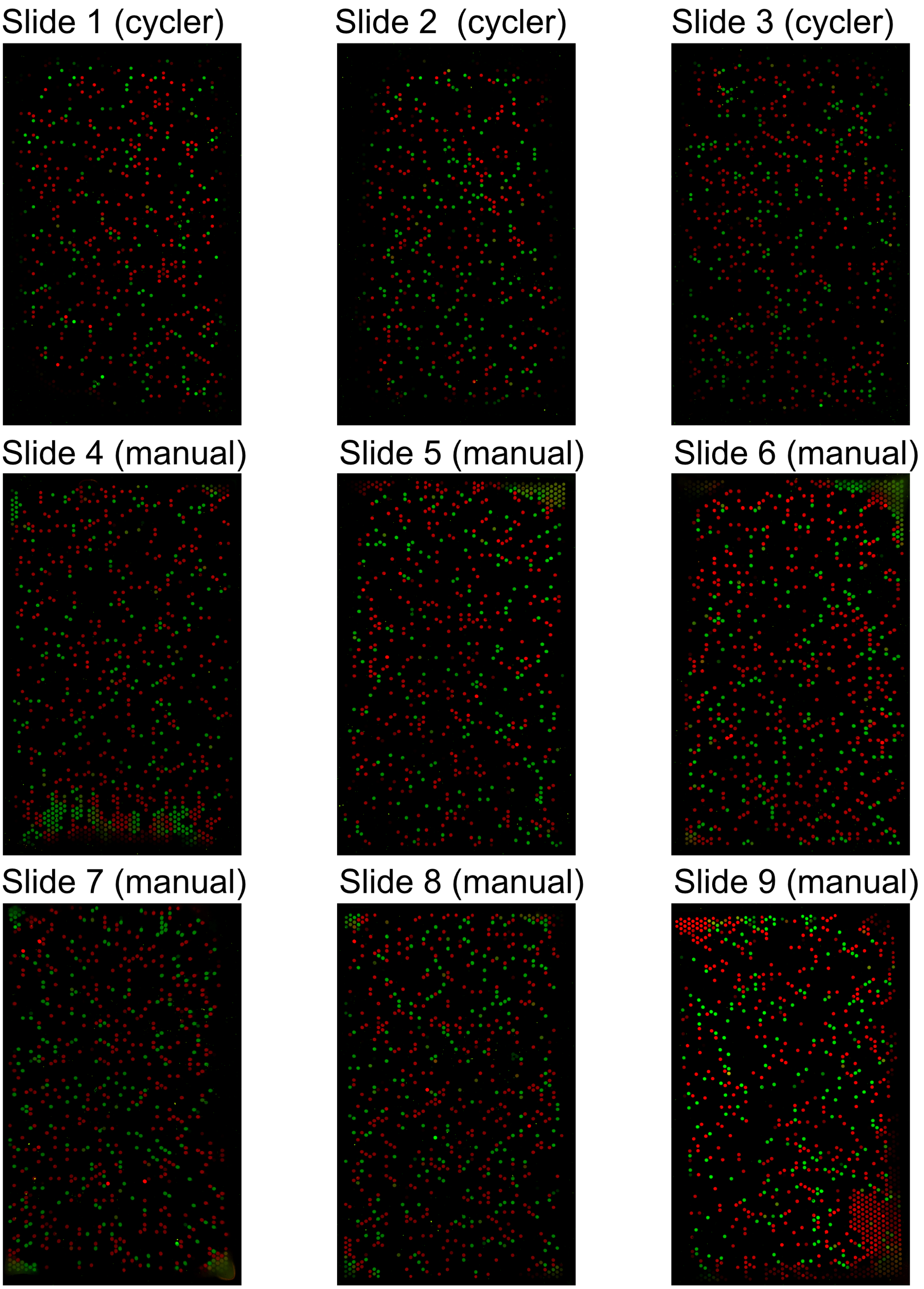
Slide 1 (cycler)
Slide 2 (cycler)
Slide 3 (cycler)
Slide 4 (manual)
Slide 5 (manual)
Slide 6 (manual)
Slide 7 (manual)
Slide 8 (manual)
Slide 9 (manual)

Slide 10 (manual)

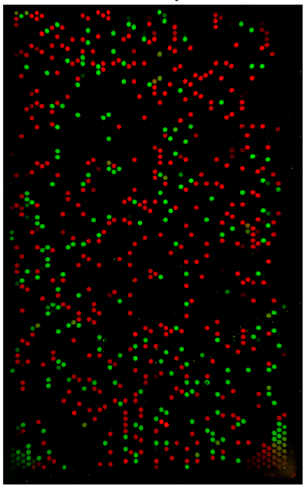

Slide 11 (manual)

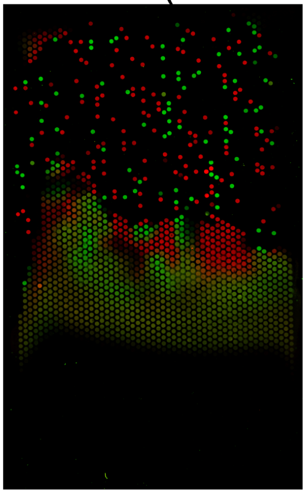

Slide 12 (manual)

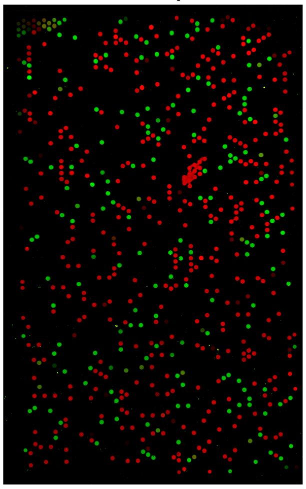

Slide 13 (manual)

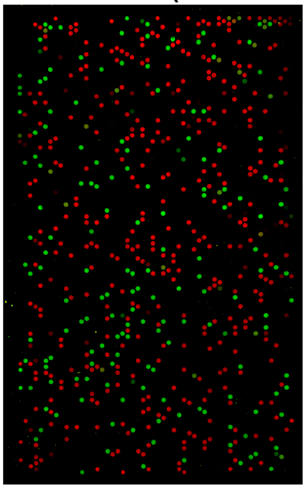

Slide 14 (cycler)

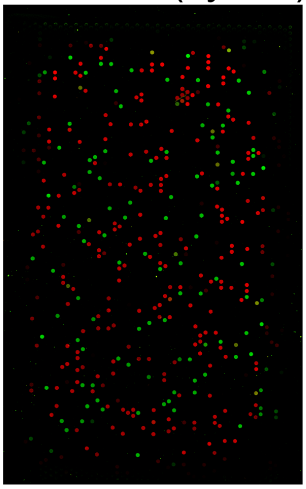

Slide 15 (cycler)

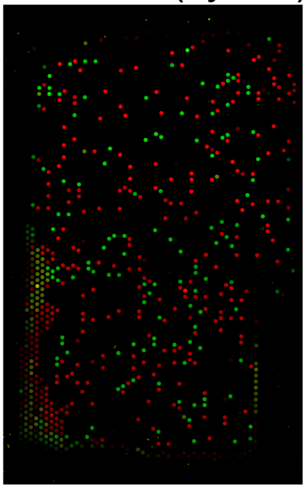

Slide 16 (cycler)

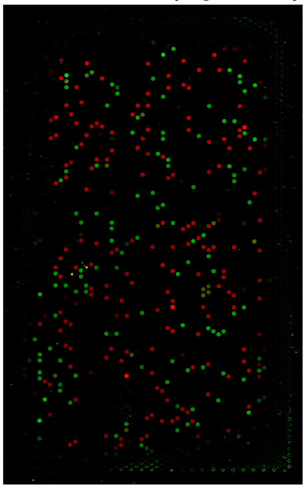

Slide 17 (cartridge)

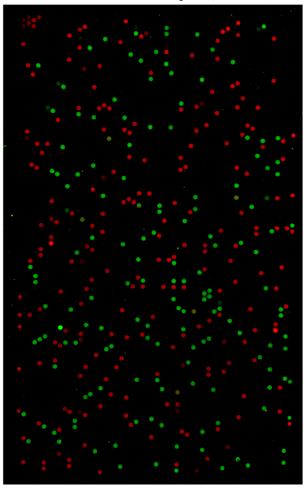

Slide 18 (cartridge)

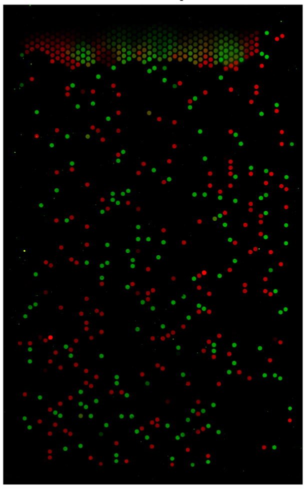

Slide 19 (cartridge)

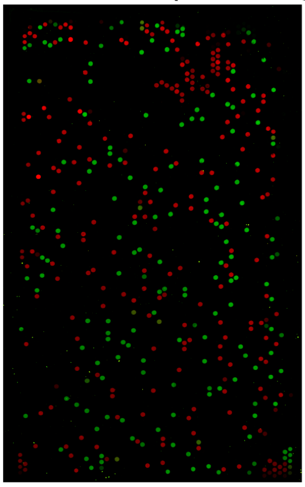

Slide 20 (cartridge)

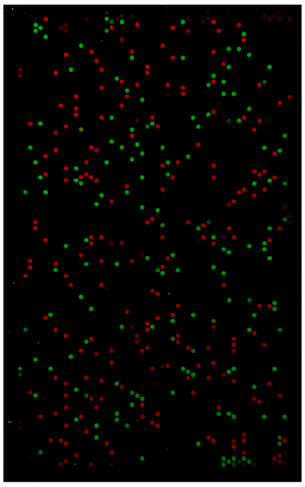

Slide 21 (cartridge)

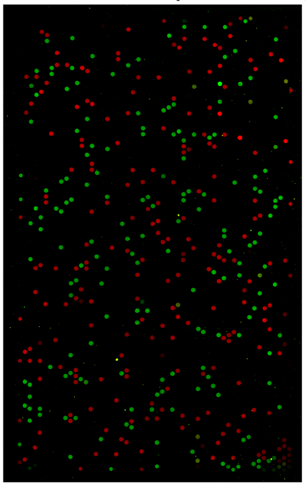

Slide 22 (cartridge)

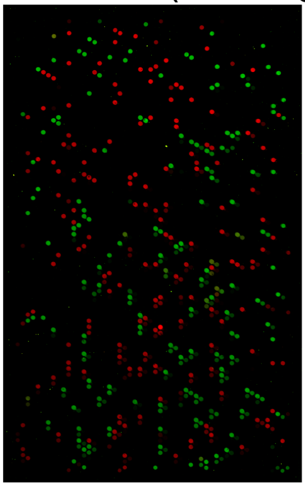

Slide 23 (cartridge)

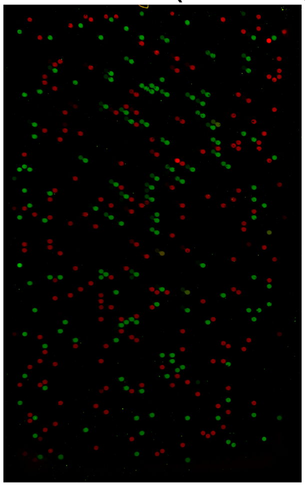

Slide 24 (cartridge)

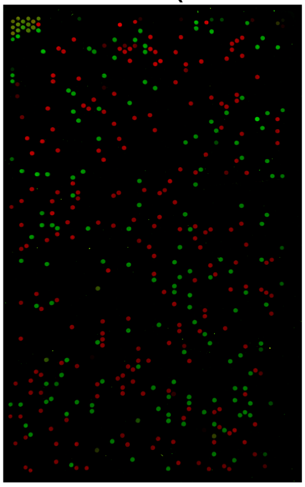

Slide 25 (cartridge)

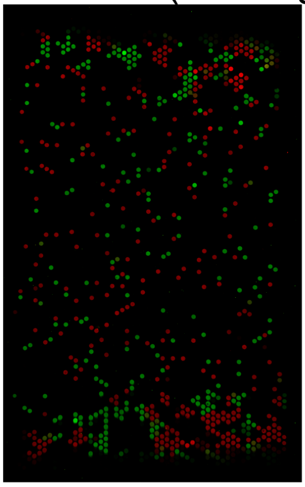

Slide 26 (cartridge)

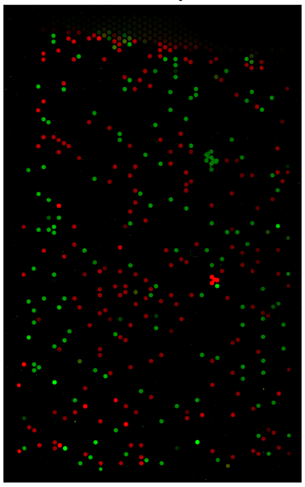

Slide 27 (cartridge)

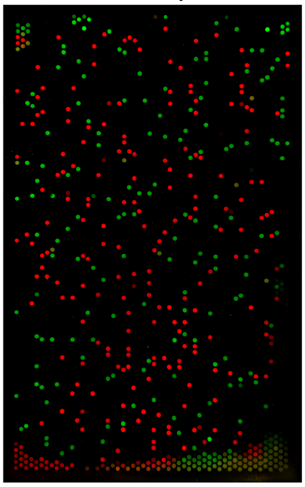

Slide 28 (cartridge)

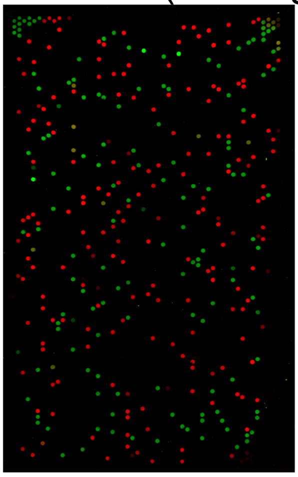

Slide 29 (cartridge)

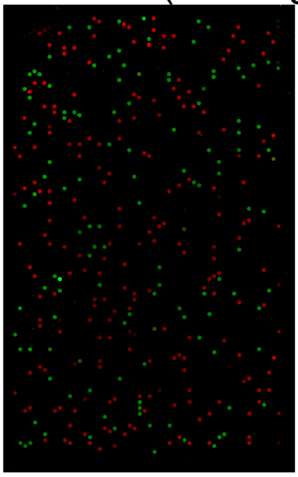

Slide 30 (cartridge)

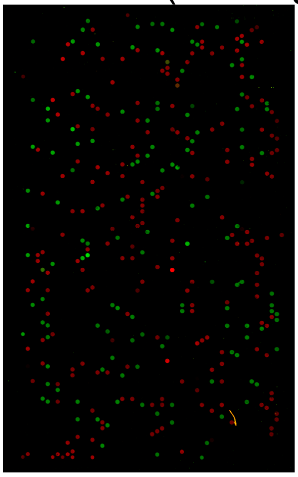

Slide 31 (cartridge)

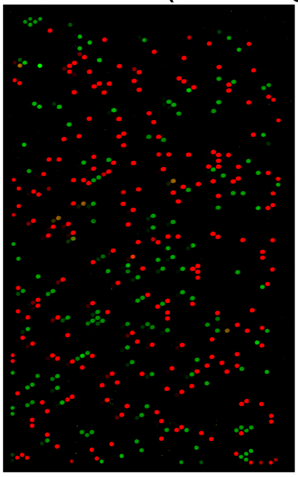

Supplementary Figure S6 Fluorescent scans of all DNA microarrays. All red channels of the images were normalized to the median of fluorescent values of the green channels.

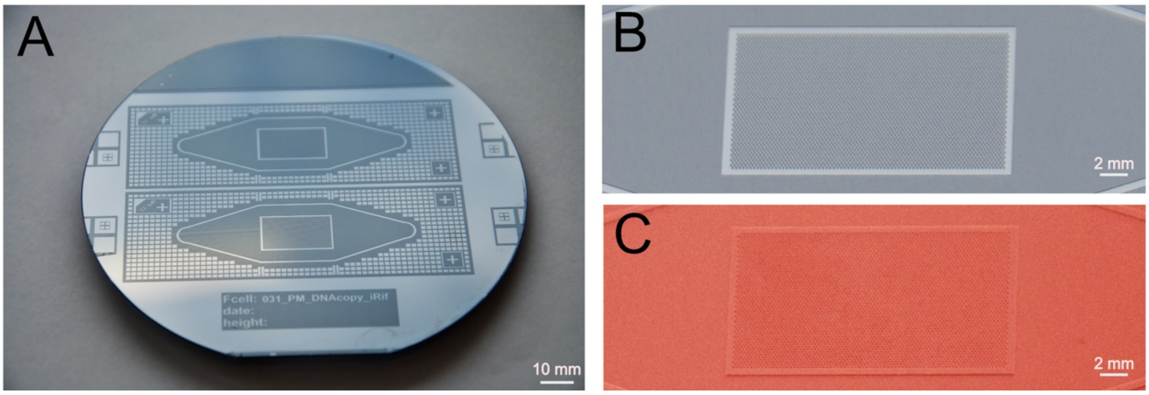

Supplementary Figure S7 Processed wafer after clean room production (A). This wafer can be used as a mold for the PDMS cavity chips. Magnification of the array region (B and C).

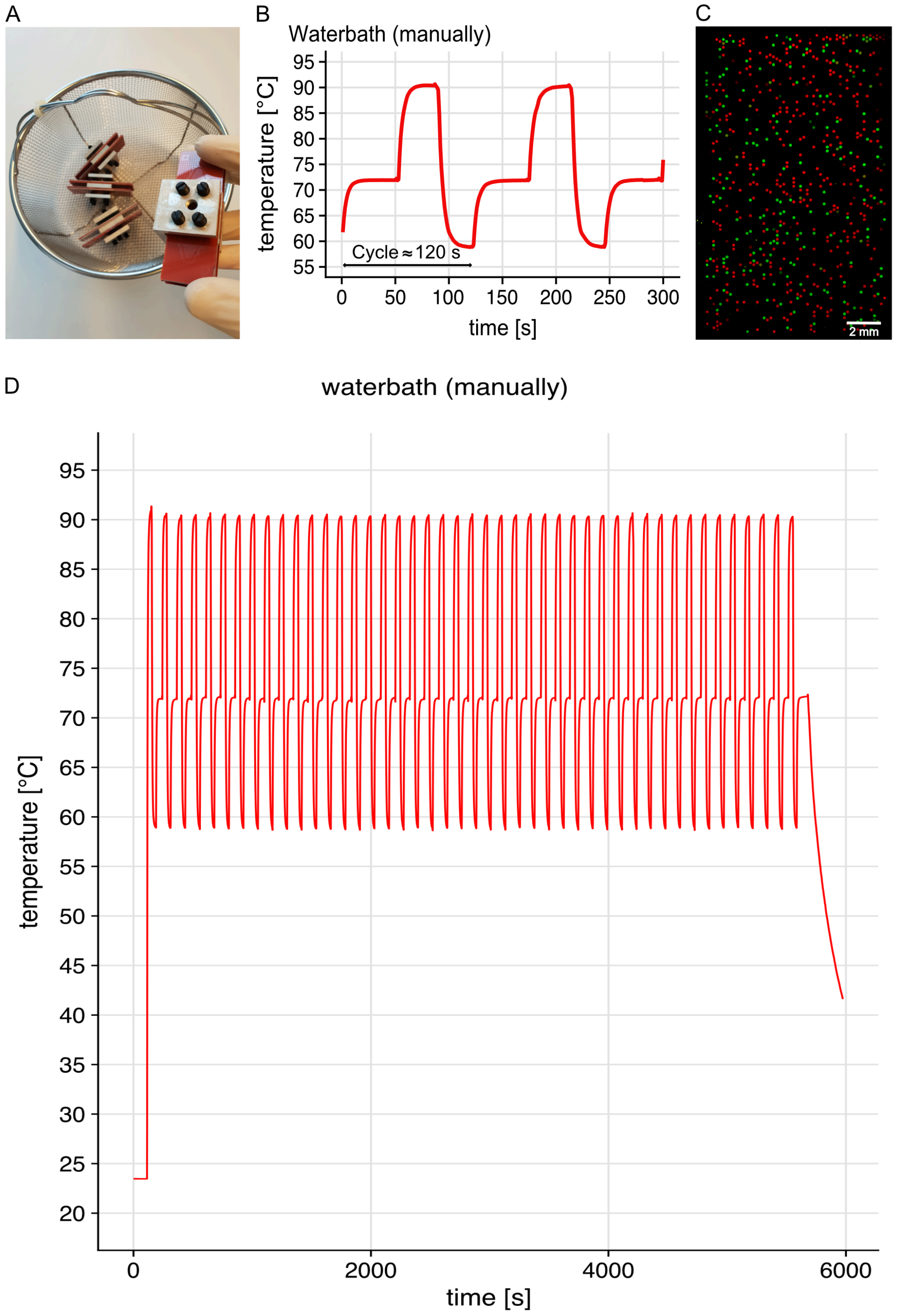

Supplementary Figure S8 Manual water bath PCR. The digitally filled and sealed PDMS cavity chips were clamped using a W-shaped aluminum holder (A). The individual holders were placed into a sieve basket to ensure a quick transfer of the chips from one water bath to the next. A representative cycle of the PCR had a duration of 120 s (B). A typical fluorescence scan of a hybridized microarray after PCR showing digitally distributed green and red DNA spots (C). In total, the complete PCR with 45 cycles had a duration of 90 min (D). The maximum heating rate from the annealing temperature to the elongation temperature step was 2.94 °C/s with an average heating rate over the first 10 s of 1.13 +/- 0.03 °C/s. The maximum heating rate from the elongation temperature to the denaturation temperature step was 4.15 °C/s, with an average heating rate of 1.57 +/-0.08 °C/s. The maximum cooling rate from the denaturation temperature to the annealing temperature step was 6.27 °C/s with an average cooling rate of -2.53 +/- 0.16 °C/s.

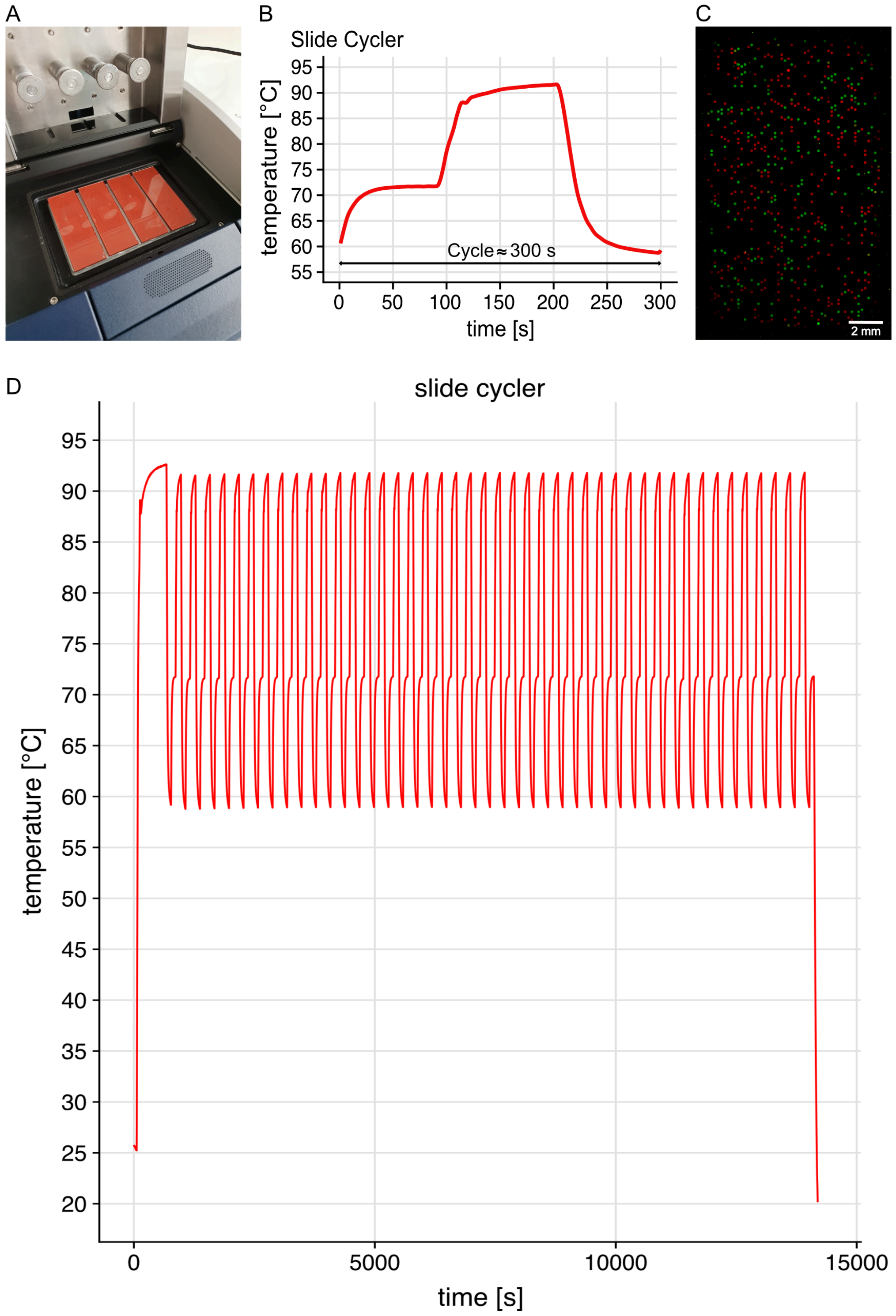

Supplementary Figure S9 Automated cycler PCR. Four digitally filled and sealed PDMS cavity chips were placed into the slide cycler (A). Here, the chips were placed upside down for better illustration. A typical cycle of the PCR with a duration of 300 s per cycle (B). A typical fluorescence scan of a hybridized microarray after PCR showing digitally distributed green and red DNA spots (C). In total the full PCR with 45 cycles had a duration of 240 min (D). The maximum heating rate from the annealing temperature to the elongation temperature step was 0.90 °C/s with an average heating rate over the first 10 s of 0.66 +/- 0.01 °C/s. The maximum heating rate from the elongation temperature to the denaturation temperature step was 1.05 °C/s, with an average heating rate of 0.7 +/-0.02 °C/s. The maximum cooling rate from the denaturation temperature to the annealing temperature step was -1.40 °C/s with an average cooling rate of -0.84 +/- 0.05 °C/s.

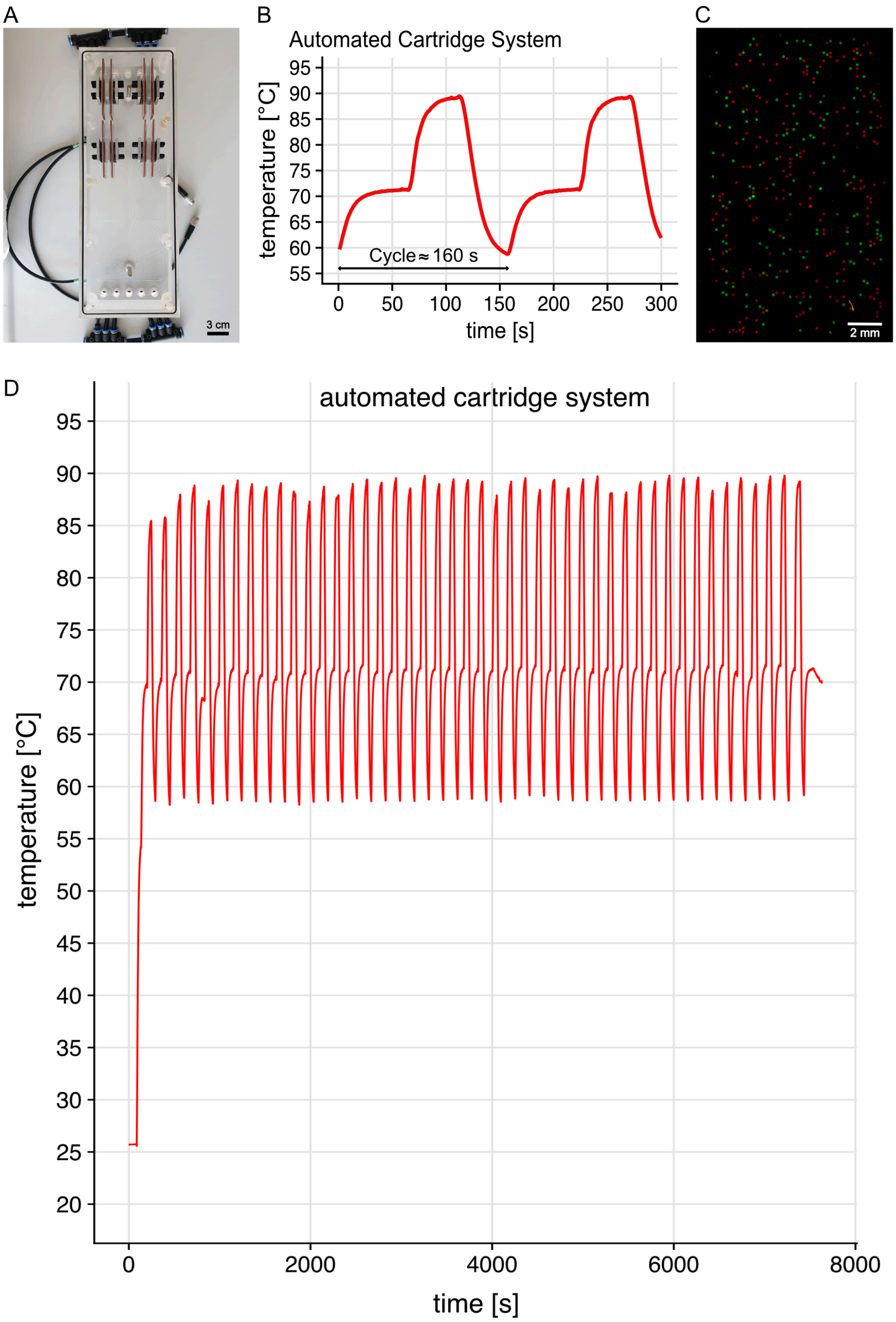

Supplementary Figure S10 Automated cartridge PCR. The digitally filled and sealed PDMS cavity chips were clamped in a W-shaped aluminum holder and placed inside of the cartridge (A). A typical cycle of the PCR with a duration of 160 s per cycle (B). A typical fluorescence scan of a hybridized microarray after PCR showing digitally distributed green and red DNA spots(C). In total the full PCR with 45 cycles had a duration of 120 min (D). The maximum heating rate from the annealing temperature to the elongation temperature step was 0.78 °C/s with an average heating rate over the first 10 s of 0.57 +/- 0.08 °C/s. The maximum heating rate from the elongation temperature to the denaturation temperature step was 1.83 °C/s, with an average heating rate of 1.09 +/-0.07 °C/s. The maximum cooling rate from the denaturation temperature to the annealing temperature step was -1.42 °C/s with an average cooling rate of -1.13 +/- 0.05 °C/s.

Supplementary Table S11 Evaluation results of DNA microarrays (Supplement Figure 6; Slide1 – Slide31). The median values of the green and red spots are fluorescence values measured with a GenePix 4000B microarray scanner using GenePix software version 7 Pro. Spots were analysed in ImageJ.

Slide ID	Method	Number of green spots	Probability of green spots	Median green spots	Median background green spots	Number of red spots	Probability of red spots	Median red spots	Median background red spots	Expected number of yellow	Observed number of yellow spots	Leakage fraction
1	cycler	171	0.045	36820	262	367	0.096	14096	88	16	7	0.061
2	cycler	199	0.049	35682	303	329	0.080	10285	89	16	15	0
3	cycler	216	0.056	31464	267	392	0.101	8230	85	22	13	0.050
4	manual	158	0.045	32656	192	359	0.102	7008	87	16	12	0.137
5	manual	184	0.047	37999	170	365	0.094	9263	86	17	12	0.051
6	manual	171	0.044	41515	170	390	0.100	10317	86	17	18	0.044
7	manual	148	0.039	27408	143	286	0.075	6117	85	11	7	0.064
8	manual	214	0.055	33577	162	417	0.108	9616	87	23	29	0.055
9	manual	189	0.053	53139	131	354	0.099	15688	87	19	9	0.121
10	manual	155	0.039	47118	133	295	0.074	13285	88	11	10	0.021
11	manual	84	0.053	44132	118	135	0.085	11344	94	7	4	0.611
12	manual	180	0.044	47644	167	427	0.105	14882	90	19	17	0.009
13	manual	197	0.048	45629	124	392	0.096	10228	83	19	17	0
14	cycler	146	0.042	41324	279	300	0.087	14757	96	13	15	0.152
15	cycler	129	0.042	42353	269	250	0.081	15146	93	10	5	0.244
16	cycler	151	0.049	35020	274	227	0.073	10998	85	11	16	0.243
17	cartridge	158	0.039	38446	152	224	0.055	11398	90	9	6	0
18	cartridge	152	0.041	37595	164	186	0.050	10393	87	8	4	0.090
19	cartridge	169	0.042	36566	167	214	0.053	8938	88	9	8	0.013
20	cartridge	155	0.039	35763	123	224	0.057	8830	87	9	8	0.032
21	cartridge	190	0.046	37598	179	228	0.056	9501	85	11	11	0
22	cartridge	129	0.032	29744	177	172	0.042	7632	89	5	6	0
23	cartridge	83	0.020	28155	87	150	0.037	6134	82	3	1	0
24	cartridge	150	0.037	30226	172	218	0.054	8645	88	8	6	0.007
25	cartridge	134	0.050	28012	179	171	0.064	6479	85	9	7	0.342
26	cartridge	140	0.038	34700	120	195	0.052	9257	94	7	8	0.087
27	cartridge	188	0.049	37520	122	212	0.055	8973	89	10	7	0.053
28	cartridge	133	0.033	35800	146	199	0.049	8837	89	7	11	0.012
29	cartridge	126	0.031	32388	103	225	0.055	6898	88	7	6	0
30	cartridge	138	0.034	30878	111	209	0.051	8186	92	7	3	0
31	cartridge	99	0.024	37369	140	159	0.039	11916	88	4	1	0

Supplementary Table S12 Evaluation of all DNA microarray spots fluorescence values of the experiment. The median values of the green and red spots are fluorescent values measured with a GenePix 4000B microarray scanner using GenePix software version 7 Pro. Spots were analysed in ImageJ.

Method	Total number of green spotsl	Overall median of green spots	Total number of red spotsl	Overall median of red spots
cartridge	2144	34582	2986	8688
cycler	1680	40127	3420	12791
manual	1012	35666	1865	14896
